# Clinical Presentation of Wide Field of Cancerization Associated with Oral Squamous Cell Carcinoma

**DOI:** 10.1155/2023/7530295

**Published:** 2023-03-17

**Authors:** Yousif Idris Eltohami, Ahmed Mohamed Suleiman

**Affiliations:** Faculty of Dentistry, University of Khartoum, Khartoum, Sudan

## Abstract

**Background:**

The late presentation of oral squamous cell carcinoma (OSCC) patients in Sudan, with advanced stages and wide field of cancerization (WFC), has a negative impact on these patients. The present study aimed to investigate the different clinical presentations of mucosal changes in WFC associated with OSCC in Sudanese patients.

**Methods:**

This a prospective longitudinal study of 93 OSCC cases. Tumor's associated field of cancerization was identified and related clinical mucosal changes were described.

**Results:**

Out of the 93 patients, 57 (61.3%) were males and 36 (38.7%) were females. Eighty-two percent of the patients presented with stage IV tumors. Ninety-two patients had multiple sites involved in the oral cavity with overlap of sites involved. The Gingivobuccal mucosa (74.2%) was the most frequent site involved. Eighty-three (89.2%) of the lesions were surrounded by mucosal changes, of them 32 (38%) surrounded by a grizzle (mixed dark and white) discoloration and 21 (26%) were surrounded by a white-smoke discoloration followed by 17 (20%) and 13 (16%) surrounded by cotton-white and Café au lait discolorations, respectively. Forty-four (47.3%) lesions had overlapping presentations and surrounded by erythematous patches.

**Conclusion:**

The present study showed that OSCC patients in Sudan present with advanced lesions, mostly associated with WFC, particularly the Toombak dippers. The different mucosal changes seen in the WFC associated with OSCC in these cases are in accordance with the known five mucosal presentations.

## 1. Introduction

Malignancies of the oral cavity are considered worldwide a major health issue. Collectively, they rank as the sixth most common cancer globally [1]. In Sudan, most of the studies on the frequency of oral cancer were institution-based studies. Salah and Mohamed [2], in a more recent study, reported that oral cancer in Sudan was in the 9th ranking position, while Elbasheir, in a previous study, showed that it was the second most common cancer in Khartoum State, accounting for 9.4% of all body cancers [3]. Cancer is a heterogeneous disease process involving the interaction of multifactorial etiopathogenesis and body vulnerability [4]. The sort of carcinogenic material, duration of tissue contact and dose, frequency, and combination of the different carcinogens; all are involved with different degrees in the process of cancer development [5]. Top on the carcinogenic materials is tobacco in its different forms of usage [6]. Toombak is a moist form of snuff, well known as a risk factor of oral squamous cell carcinoma (OSCC) in Sudan. It is made from a tobacco species with a high level of nicotine and minor alkaloids, added to it Atron (sodium bicarbonate), and the blend is mixed with water to become moist and used after a period of time [7]. The hypothesis of wide field of cancerization (WFC) by Slaughter et al. [8] stated that the whole epithelial surface of the upper aerodigestive tract has an increased risk for the development of malignant and potentially malignant lesions because of multiple genetic aberrations in the entire affected tissue area [9]. The term WFC was used to describe (pre-)neoplastic processes at multiple regions, which may develop independently. The presence of multiple primary and secondary tumors could be due to a wide exposure of the epithelial cells at different sites to carcinogens or to the recent proposition that it is due to a lateral wide migration of preneoplastic epithelial cells which subsequently may give rise to multiple clonally related lesions [8, 10]. Recently, it is becoming known that genetically altered cells could spread widely across the OSCC epithelium into clinically and histologically normal tissue, which could elucidate the concept of the field alteration of cancer [10]. One of the known clinical characteristics of wide field of oral cancerization is the wide involvement by OSCC at multiple oral cavity sites. Clinically, the “Snuff Dippers' Lesions” present as varieties of presentations ranging from wrinkling to different discolorations associated with different mucosal thickening [11]. In the last decade, Suleiman reported five different presentations of clinical mucosal changes at the site of Toombak dipping among Sudanese dippers: Presentation I: wrinkling with slight discoloration, Presentation II: Café au lait discoloration (brownish discoloration), Presentation III: white-smoke discoloration, Presentation IV: grizzle (a mixture between white and dark discoloration), and Presentation V: cotton-white discoloration [6]. Recently, Abdalla et al. [12] described in their clinical examination of their patients the predominance of “Presentation I” in 43.5% of the cases, followed by Presentations: III and II accounting for 16.1% and 14.9%, respectively. The present study is aimed to describe the different clinical presentations and mucosal changes of WFC of OSCC patients in Sudan.

## 2. Material and Methods

**Figure 1:**
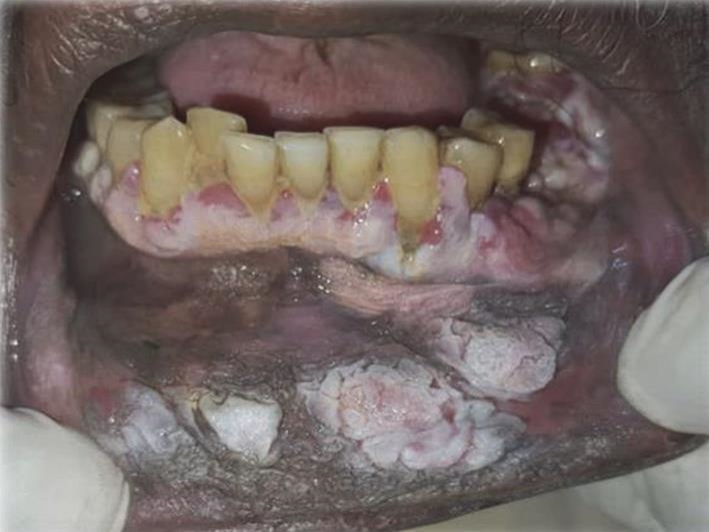
Patient of OSCC associated with different mucosal changes of wide field of cancerization (grizzle and cotton-white presentations).

**Figure 2:**
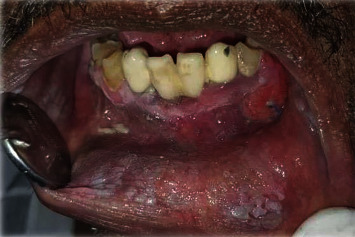
Patient of OSCC with wide field of cancerization (white-smoke discoloration).

Prospective longitudinal hospital-based study carried out at Khartoum Teaching Dental Hospital (KTDH). The main hospital and center of oral and maxillofacial surgical oncology in Sudan. The samples were selected after thorough clinical examination of the oral cavity and head and neck regions for clinically suspicious ulcerative lesions, which were associated with wide field change of the oral mucosa (Figures 1 and 2). Patients presenting at the time of the study having recurrent or second primary lesions and patients who received neoadjuvant radiation and/or chemotherapy were excluded from the study.

## 3. Statistical Analysis

Data were entered in a computer master sheet using SPSS version 26. Statistical analysis was set at 95% confidence level, 0.2, the width of the confidence interval, and the level of significance alpha is 0.05. Chi-square test was used to test the difference between categorical variables, and Fisher's exact test was used as an alternative test in case of sparse data. Multivariate analysis and logistic regression were performed for better prediction precision and to control confounders, and both were interpreted in the form of odd ratio and *P* value. Descriptive statistics were conducted for variables: age, site, TNM staging, etc.

## 4. Results

The present study is a prospective longitudinal hospital-based study. The material were patients of OSCC operated at KTDH in the period: November 2018 to April 2022. The study included 93 patients (186 specimens) with OSCC who were diagnosed and surgically treated at KTDH. The patients were 57 (61.3%) males and 36 (38.7%) females. The mean age of the patients at the initial diagnosis was 57.52± years with a standard deviation of 12.506 and a minimum of 25 and maximum of 80 years. Regarding the social habits, 38 (39.9%) of the patients were snuff dippers, 19 (20.4%) were tobacco smokers, and 14 (15.1%) were alcohol drinkers (Table 1). The mean age of commencement of snuff dipping was 23.25 ± standard deviation of 5.36 with a minimum age of commencement of 10 and a maximum of 40 years. The mean duration per day for snuff dipping was 4.55 ± standard deviation of 1.032 with a minimum of two and a maximum of eight times per day. The mean age of smoking commencement for the participant was 19.88 ± standard deviation of 6.470 with a minimum age of 7 and a maximum age of 50 years. The mean quantity of smoking in packs per day was 2.18 ± standard deviation of 0.529 with a minimum of one pack and a maximum of three packs per day. The mean duration of smoking per day was 5.38 ± standard deviation of 1.088 with a minimum of three and a maximum of seven times. The mean age of commencement of alcohol was 27.92 ± standard deviation of 3.43 with a minimum of 20 and a maximum of 30 years. The mean quantity of alcohol in cups per day was 3.67 ± standard deviation of 0.985 with a minimum of two and a maximum of five cups per day. The duration per day of alcohol was 2.25 ± standard deviation of 452 with a minimum of two and a maximum of three times per day. All alcohol drinkers were males. In this study, 66% of the patients showed poor oral hygiene.

### 4.1. Sites of the Lesions

Ninety-two patients had multiple sites involved in the oral cavity with overlapping in the presentations and sites involved. The gingivobuccal mucosa and gingivolabial mucosa were the most frequent sites of OSCC associated with WFC; 69 (74.2%) and 58 (62.3%), respectively. The tongue and the floor of the mouth were involved in 23 (24.7%) and 26 (28%) patients, respectively. The palate was the least site involved where only eight (8.6%) cases were recorded in the series (Table 2). According to the side of occurrence, the left side was involved in 41 (44.1%), the right side was involved in 34 (36.6%), and in 18 (20.4%) patients, there was bilateral involvement. In 80 (86.0%) patients, the lesion was in the anterior region, and in 72 patients (77.4%) the involvement was in the posterior region (Table 3).

### 4.2. Clinical Presentations

**Figure 3:**
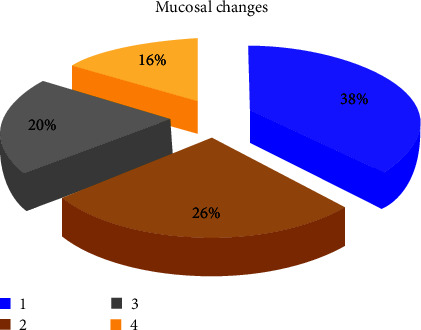
Distribution of mucosal changes.

Regarding the clinical presentation of OSCC lesions in the present study, all the 93 (100%) cases presented with an ulcer as a complaint; 83 (89.2%) of the lesions were surrounded by mucosal changes; of them, 32 (38%) were associated with a grizzle (mixed dark and white) discoloration (Figure 1), 21 (26%) were associated with a white-smoke discoloration (Figure 2), 17 (20%) were associated with a cotton-white discoloration, and the remaining 13 (16%) were associated with a Café au lait discolorations (Figure 3).

Also, of the overlapping presentations, 44 (47.3%) of the lesions were associated with erythematous patches, which highlighted the clinical presentation of a wide field of changes. Swelling and pain were reported among 18 (19.4%) of the patients, and numbness was reported by 22 (23.7%) of them (Table 4). The mean ulcer duration in months was 5.23 ±standard deviation of 1.075 with a minimum of 3 and a maximum of 8 months.

### 4.3. Clinical Characteristics of the OSCC Ulcer

The characteristics of these malignant ulcers were as follows: 80 (86.0%) of the ulcers had everted edges and 13 (14.0%) had rolled edges. Most of the margins, 90 (96.8%), were irregular. The ulcer base characteristically had induration in 92 (98.9%) of the cases. Forty-one (44.15%) of the cases had fixation to the underlying anatomical structures. More than half of the lesions (57.0%) were endophytic and 40 (43.0%) were exophytic (Table 5). Fifty-four (58.1%) of the cases had ulcers measured around 45 mm. The smallest recorded diameter was 25 mm, which was reported in only one case. Only one case in the present study showed the largest recorded diameter of 70 mm (Table 6).

### 4.4. Clinical TNM Staging

Depending on the TNM staging AJCC the 8 edition, in the present study, of the cases, 71 (76.3%) patients presented with an advanced tumor size T4a, 19 (20.4%) cases presented with a tumor size T3, two cases (2.2%) presented with an advanced tumor size T4b, and one case (1.1%) presented with a tumor size T2. At the initial diagnosis, 68 (73%) patients presented with (N2) nodal involvement, 38 (40.9%) of them had N2a stage, and 22 (23.7%) had N1 early nodal involvement. The advanced N3 nodal disease was found in three (3.2%) of the cases. All the cases (93%–100%) showed no distant metastasis (M0) (Table 7). Of the series, 69 (74.2%) patients presented with advanced stage IVa (T4a + N0/N1/N2 + M0) tumors, and seven (7.5%) patients presented with stage IVb. Additionally, 17 (18.2%) patients presented with stage III tumors, and there were no cases of early cancer, i.e., stage I and stage II among the series (Table 8).

### 4.5. Associations

1. Toombak-dippers have multiple sites involved more frequently, while non-Toombak-dippers tend to have single site involved; this difference was found to be significant (*P* value = 0.02). No significant association between smoking and number of sites involved (*P* value = 0.173).
2. Toombak-dippers their lesions were more frequently stage IV (T4a + N0/N1/N2 + M0) cases; the difference between them and the nondippers was found to be significant (*P* value = 0.039). Patients who presented with white (cotton-white discoloration) patches were significantly at stage IVA (T4a/N0/N1/N2 + M0) (*P* value = 0.001).

#### 4.5.1. Crosstabulations

Around 73.6% of the male patients had bad social habits and only 8% of the female patients reported bad social habits. Sixty-five percent of the males were snuff dippers and smokers, and 30% of them were smokers. For the females 2% were snuff dippers and 5% were smokers. Eighty-four percent of the snuff dippers had multiple sites involvement of OSCC, and 94% of the cases had their primary in the anterior gingivolabial region in correlation to the site of dipping. All OSCC lesions of the snuff dippers presented clinically as an ulcer. Ninety-five percent and 71% of the snuff dipper patients had a broad spectrum of mixed white and dark discoloration and erythematous patches in their clinical presentation respectively. Eighty percent of the male patients presented late in stage IVa and the females in this advanced stage were of relatively lower percentage (63%). Ninety-two percent of the snuff dippers presented in the advanced stage IVa, and also 85% of the patients with white patches presented clinically with advanced and very advanced stages IVA and IVB.

## 5. Discussion

Multistep carcinogenesis is one of the most breaking through concepts in pathogenesis of cancer. It highlights the process by which the progression from a normal cell to a cancer cell by accumulation of genetic aberrations occurs [9]. Genetically mutated cells have the ability to spread vigorously beyond the OSCC epithelium, into clinically and histologically normal tissues, which may clarify the wide field alteration of cancer [10]. The study is designed to throw more lights on oral cancer in Sudan by its paramount analysis of the different clinical presentations of mucosal changes of WFC associated with OSCC. In this study, the mean age of the patients at the initial diagnosis was 57 years. The literature showed that the vast majority of OSCC cases worldwide occur in the fifth to the eighth decade of life [13, 14]. Similar to our result, the other previous Sudanese studies reported a median age of 53–60 years at the initial presentation [3, 6, 15]. Although the last two decades showed a noticeable involvement in the young age groups, as reported by Liu et al. [16], worldwide incidence rates of oral cancer differ dramatically between both genders due to different environmental and social factors. The present study showed a male gender predilection with a male-to-female ratio of 1.6 : 1. Similarly, other studies showed a predominance of males, with a male-to-female ratio ranging from 2 : 1 to 4 : 1 [17, 18]. In the present study, 39.9% of the cases were Toombak dippers, 20.4% were cigarette smokers, and 15.1% were alcohol drinkers. The mean age of commencement of Toombak dipping was 19 years. Alim [19] reported similar findings with slightly higher percentages, as 53.5% of their patients were Toombak-dippers, 27.1% were tobacco smokers, and 11.4% were alcohol drinkers. Different results were stated by Fang et al. [20], who reported that 61.3% of their patients were smokers and 38.8% had a history of alcohol consumption. One of the known clinical characteristics of wide field of oral cancerization is the wide involvement by OSCC at multiple oral cavity sites. The dominance of males in these studies is attributed to the habit of Toombak dipping, which is more popular among Sudanese males. On the other hand, Ferlay et al. [21] reported that in Melanesia, there is a high incidence of OSCC among both females and males without mentioning any risk factor. Some authorities attributed the etiology of cases of unknown risk factors to the human papillomavirus (HPV), despite the fact that most HPV-associated SCCs are of an oropharyngeal origin [22]. The present study showed that 99% of the cases had involved multiple sites, and only one case had involved single site. In contrast to the previously published studies [6, 23, 24], which showed the gingivolabial site as the most frequent site for OSSC in Sudan, the present study showed the gingivobuccal mucosa was the commonest involved site accounting for 74.2% of the cases, followed by the gingivolabial mucosa accounting for 62.3%. This variation may be attributed to the nature of the wide field involvement of multiple sites in almost all the cases found in the present study. The involvement of the buccal mucosa in most of the cases is most probably due to the lateral spread of the malignant cells and to its wide exposure to the carcinogenic substances. The clinical outcome of the multiple involvement of wide field of cancerization in Toombak dippers, could be attributed to the chemical composition as well as the physical nature of Toombak.

In the present study, the commonest clinical presentation of OSCC lesions was an ulcer in 93 (100%) of the cases. The ulcer was usually (89.2%) associated with discoloration of the surrounding mucosa. In 32 (38%) cases, the associated discoloration was grizzle (mixed dark and white), in 21 (26%) cases, it was white-smoke discoloration, and in rest 17 (20%) cases, it was a cotton-white discoloration. These presentations highlight the clinical characteristics of the wide mucosal alterations among Toombak-dippers as was described previously by Suleiman [6] and recently by Abdalla et al. [12], who reported that clinical examination of the dipping sites of 161 Toombak users and showed the followings: wrinkling with/without slight discoloration was seen in 70 (43.5%). White-smoke discoloration was seen in 26 (16.1%) cases. Cafe au lait discoloration, cotton-white patches, and grizzle (mixed white and deep patches) discoloration were seen in 24 (14.9%), 21 (13%), and 20 (12.4%) of the cases, respectively. The variation from the present study was probably due to the fact the cases in the present study were diagnosed OSCC cases, which were associated with advanced presentations of mucosal changes as compared to the cases in the study of Abdalla et al. [12], which were diagnosed at out-door oral cancer screening camps. It is worth mentioning that Abdalla et al. [12] had some cases diagnosed with hyperkeratosis to mild and severe dysplasia without frank carcinoma. Axéll et al. [25] studied the clinical presentations of the Swedish snus-dippers and described it in four grades. Their descriptions are in close resemblance to what was reported by Suleiman [6], but with slight modifications in the clinical characteristics. These differences may be to chemical nature of the snuffs used or due to the variations in the processing methods of the material in each country.

Russo et al. [26] reported in their case series that OSCC lesions have presented clinically as an ulcer (69.7%), red and white patches (45.4%), exophytic mass (12.1%), and more than two-thirds of them were painless, regardless of their clinical presentation. Many studies on Sudanese OSCC patients reported that the vast majority of these patients presented with an indurated exophytic ulceration in the gingivolabial region where Toombak was dipped. Other clinical presentations included dysphagia, earache, and trismus [6]. In the present study, more than half of the lesions (57.0%) were endophytic, with 58% of the cases have a size of 45 mm of malignant tissues, which may influence the clinical involvement of the WFC in these cases. Pires et al. [27], in their cohort study of 346 OSCC patients, reported that the size of OSCC ulcers was ranging from 10 to 100 mm with a mean size of 34 mm. In the present study, 76.3% of the patients presented with an advanced tumor size T4a. Similar findings were reported by Oliveira et al. [28], who reported in their longitudinal study that 82.1% of their patients had advanced-stage (III/IV) OSCC. Elaiwy et al. [29], from Qatar, reported that early T1 and T2 lesions comprised about two-thirds of the cases in 39.8% and 26.2% of their patients, respectively. They stated that in Qatar, there is a meticulous cancer referral system that is attentive for any suspicious malignant or premalignant changes. Different from the findings of the present study, Ahmed et al. [30] reported that only 25.6% of their cases had regional neck metastasis, while 74.4% had no evidence of nodal involvement. The findings of the study regarding late presentation of the OSCC were in agreement with Osman et al. [15] stated that 94% of their Sudanese patients presented in stage IV. Similarly, Zargoun et al. [31] in their study reported that the majority (69%) of their patients presented in stage IV. The late presentation of oral cancer patients in Sudan is due to many factors, including lack of awareness, the presence of a weak health system in the country, and an ailing economy of the state. All these factors contribute to a delay from the patient's side as well as from the health personnel's side. Local and locoregional recurrences as a clinically significant outcome of OSCC associated with WFC were reported by Eltohami and Sulaiman [32] in lesions associated with mucosal changes, particularly in Toombak snuff dipper patients.

## 6. Conclusion

Toombak dipping is the most frequently reported risk habit of OSCC, followed by cigarette smoking. The majority of the patients in the present study had advanced stage IV tumors associated with WFC on the initial presentation. The commonest mucosal change of the WFC was grizzle (mixed dark and white) discoloration, commonly followed by white-smoke and cotton-white discolorations.

## Figures and Tables

**Table 1:** Bad risky habits.

	Frequency (%)
Bad habit	
Smoking	19 (20.4%)
Alcohol	14 (15.1%)
Toombak dipping	38 (39.9%)
Type of smoking	
Tobacco smoking	18 (19.4%)
Water pipe—shisha	5 (5.4%)
Type of alcohol	
Marisa	12 (13%)
Liquor	1 (1.1%)

**Table 2:** Oral cavity subsites.

Site	Frequency (%)
Gingivobuccal mucosa	69 (74.2%)
Gingivolabial mucosa	58 (62.3%)
Floor of the mouth	26 (28%)
Tongue	23 (24.7%)
Maxillary sinus	19 (20.4%)
Retromolar trigone	10 (10.8%)
Palate	8 (8.6%)

**Table 3:** Side distribution of the lesions.

	Frequency (%)
Side of the region	
Right side	34 (36.6%)
Left side	41 (44.1%)
Bilateral	18 (20.4%)

**Table 4:** Clinical presentations.

	Frequency (%)
Ulcer	93 (100%)
Swelling	18 (19.4%)
Ulcers associated with different mucosal changes	83 (89.2%)
Ulcers associated with by erythematous patches	44 (47.3%)
Pain or numbness	22 (23.7%)

**Table 5:** Ulcer description.

	Frequency (%)
Edges	
Everted	80 (86.0%)
Rolled	13 (14.0%)
Margins	
Irregular	90 (96.8%)
Inflamed	3 (3.2%)
Base description	
Induration	92 (98.9%)
Fixation	41 (44.15%)
Surface description	
Endophytic	53 (57.0%)
Exophytic	40 (43.0%)

**Table 6:** Ulcer size in mm.

	Frequency (%)
Ulcer diameter size (mm)	
45	54 (58.1%)
50	34 (36.5%)
60	3 (3.2%)
25	1 (1.1%)
70	1 (1.1%)

**Table 7:** TNM staging.

	Frequency (%)
Tumor staging	
T4a	71 (76.3%)
T3	19 (20.4%)
T4b	2 (2.2%)
T2	1 (1.1%)
Lymph node involvement	
N2a	38 (40.9%)
N2b	27 (29.0%)
N1	22 (23.7%)
N3	3 (3.2%)
N2c	2 (2.2%)
Metastasis	
M0	93 (100%)

**Table 8:** Overall tumor staging.

	Frequency (%)
Staging	
Stage IVa (T4a + N0/N1/N2 M0)	69 (74.2%)
Stage III (T3 + N0/N1 + M0)	14 (15.1%)
Stage IVb (any T + N3 + M0)	4 (4.3%)
Stage IVb (T4b + any N + M0)	3 (3.2%)
Stage III (T1/T2 + N1 + M0)	3 (3.2%)

## Data Availability

The datasets used and/or analyzed during the current study are available from the corresponding author upon reasonable request.

## Additional Points

*Medical Registration Reference of the Ethical Clearance.* FM\DO\EC, ethical clearance was uploaded.
